# Brønsted acidic ionic liquids for cellulose hydrolysis in an aqueous medium: structural effects on acidity and glucose yield†

**DOI:** 10.1039/c8ra01950a

**Published:** 2018-04-18

**Authors:** Shiori Suzuki, Yuko Takeoka, Masahiro Rikukawa, Masahiro Yoshizawa-Fujita

**Affiliations:** Department of Materials and Life Sciences, Sophia University 7-1 Kioi-cho, Chiyoda-ku Tokyo 102-8554 Japan masahi-f@sophia.ac.jp

## Abstract

The conversion of cellulose into valuable chemicals has attracted much attention, due to the concern about depletion of fossil fuels. The hydrolysis of cellulose is a key step in this conversion, for which Brønsted acidic ionic liquids (BAILs) have been considered promising acid catalysts. In this study, using BAILs with various structures, their acidic catalytic activity for cellulose hydrolysis assisted by microwave irradiation was assessed using the Hammett acidity function (*H*_0_) and theoretical calculations. The glucose yields exceeded 10% when the *H*_0_ values of the BAIL aqueous solutions were below 1.5. The highest glucose yield was about 36% in 1-(1-octyl-3-imidazolio)propane-3-sulfonate (Oimps)/sulfuric acid (H_2_SO_4_) aqueous solution. A long alkyl side chain on the imidazolium cation, which increased the hydrophobicity of the BAILs, enhanced the glucose yield.

## Abstract 2

Using Brønsted acidic ionic liquids with various structures, their acidic catalytic activity for cellulose hydrolysis assisted by microwave irradiation was assessed using the Hammett acidity function (*H*_0_) and theoretical calculations.

## Graphical abstract

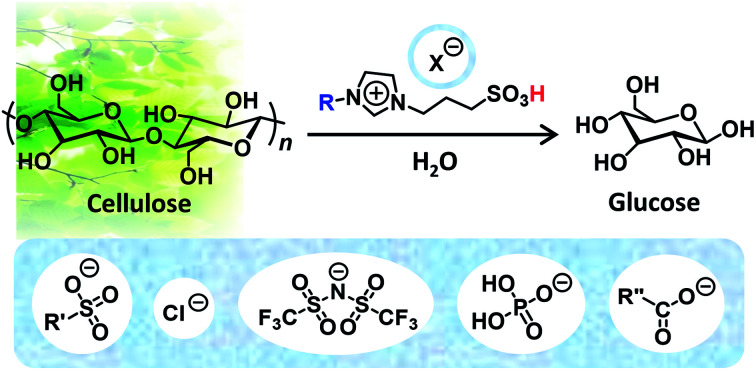

## 1. Introduction

Cellulose is the most abundant natural polymer consisting of glucose units linked *via* β-1,4-glycosidic bonds to form linear molecular chains.^1^ The hydrolysis of cellulose to fermentable sugars is an essential step for the production of bioethanol, which is expected to be a source of renewable energy. Through the hydrolysis step, cellulose is firstly degraded to oligosaccharides, and then glucose is yielded in further depolymerisation reactions. Generally, glucose can isomerise to fructose under Lewis acid catalysts, and the subsequent dehydration generates 5-hydroxymethylfurfural (HMF) and other valuable chemicals. However, this series of chemical conversion processes of cellulose is difficult, due to the strong inter- and intramolecular hydrogen bond network of the cellulose chains. Therefore, the degradation of cellulose in the first step requires drastic pretreatments at high pressure and temperature before the hydrolysis step.^2^ More efficient, simpler, and greener technologies are needed to produce cellulosic ethanol and other useful substances.

**Scheme 1:**
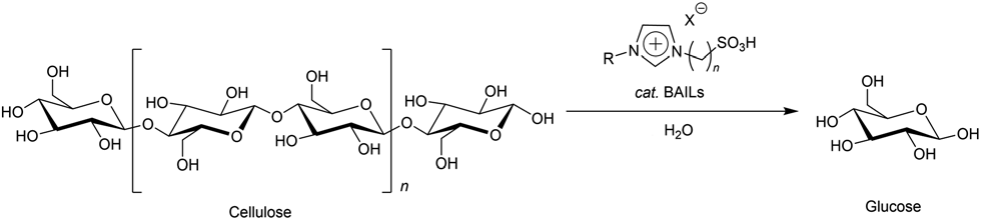
Cellulose hydrolysis to glucose using Brønsted acidic ionic liquids as both solvent and acidic catalysts.

Ionic liquids (ILs), which contain excellent hydrogen bond acceptors such as Cl and acetate (OAc) anions, have been utilised to dissolve cellulose^3^ and convert it into valuable substances.^4–6^ Due to their thermal stability and recyclability,^7–9^ ILs are known as green solvents for cellulose-related applications. In addition, there is a high degree of flexibility in their design, and various types of ILs with tailored functionalities have been discovered. Recently, Brønsted acidic ionic liquids (BAILs) have attracted the attention of researchers.^10–12^ BAILs are ILs functionalised to have acidic catalytic ability in addition to cellulose-dissolution ability, thus they behave as both the solvent and catalyst in the cellulose hydrolysis (Scheme 1). The use of BAILs in place of conventional acid catalysts has several advantages, including freedom from the need to neutralise and separate the acid catalysts after the reaction. Namely, there is no acidic waste through the whole process.^13^ The first application of BAILs to cellulose hydrolysis was reported by Amarasekara and Owereh in 2009.^14^ In that work, BAILs functionalised with SO_3_H groups could work as solvents as well as acidic catalysts for cellulose hydrolysis under moderate conditions at 70 °C for 30 min, to yield 62% of the total reducing sugars (TRS) after 1 h of preheating without water. Those authors showed that a higher concentration of the SO_3_H active sites in the BAILs accelerated the reaction and lowered the operating temperature, reducing the energy cost. The same research group also studied the effect of the cation of BAILs on the acidic catalytic ability for cellulose hydrolysis. They demonstrated that BAILs with imidazolium cations had higher acidic catalytic ability than those with pyridinium and triethanol ammonium cations.^15^ Parveen and Upadhyayula investigated the correlation between the acidity of BAILs with different acidic functional groups (SO_3_H, COOH, and OH) and the TRS yields in cellulose hydrolysis with the combined use of 1-butyl-3-methylimidazolium chloride ([Bmim]Cl). Their results showed that BAILs with SO_3_H groups had the highest acidity and catalytic activity, with 85% TRS yield at 100 °C after 90 min.^16^

After gradual development for a decade, some BAILs have shown higher acidic catalytic activity than Brønsted acids for cellulose hydrolysis under certain conditions.^11,14,17^ Amarasekara and Wiredu demonstrated the superior catalytic activity of a BAIL, 1-(1-methyl-3-imidazolio)propane-3-sulfonate/hydrochloric acid (Mimps/HCl), compared to H_2_SO_4_ in cellulose hydrolysis by a conventional heating method. In their report, while measurable catalytic activity of H_2_SO_4_ was observed for glucose generation (<17%), Mimps/HCl performed better (glucose generation <22%) at a relatively low temperature range from 140 °C to 170 °C. However, the glucose yield still remained at 22% even after 3 h of treatment at 170 °C, so further improvements are required.^15^ On the other hand, Kuroda *et al.* reported that microwave heating effectively assisted the hydrolysis reaction using a BAIL catalyst, leading to 40% glucose yield after only 12 min at 160 °C,^18^ owing to the characteristic strong microwave absorption of ILs.^19,20^ Thus, it was suggested that microwave heating of the BAILs might lead to higher glucose yield.

Beyond the acid hydrolysis reaction, a large variety of BAILs have been investigated as acid catalysts for multiple chemical reactions.^14,15,21,22^ The Hammett acidity function (*H*_0_) has been widely used as an index to evaluate the Brønsted acidity of BAILs. According to the reported *H*_0_ values,^16,23,24^ a shorter spacer length between the acidic functional group and cation and a larger number of acidic protons increase the proton donor ability of BAILs. It was also reported that the HSO_4_ anion showed higher Brønsted acidity in water than the Cl anion.^24^ However, the detailed effect of the BAIL structure (*e.g.* anion species and side chain length of the cations) on *H*_0_ has not been clarified. Moreover, there is no confirmation that the *H*_0_ directly indicates the acidic catalytic activity of BAILs, especially in terms of the glucose yield through cellulose hydrolysis.

Based on this background, a comprehensive study of the *H*_0_ of BAILs and the corresponding glucose yields would enable a better understanding of the factors contributing to their acidic catalytic activity and also the designing of BAILs for future applications. In this study, BAILs with different structures of the anion species and the alkyl spacer, and side chain lengths in the cations were synthesised and used as acid catalysts for cellulose hydrolysis assisted by microwave irradiation in an aqueous medium. The detailed relationship between the acidic catalytic activity of the BAILs and the glucose yield was firstly investigated using the *H*_0_ determined from UV-vis spectroscopy. In addition, IR spectroscopy analysis and theoretical calculations using density functional theory (DFT) optimisation were subsequently conducted to further examine the structural effects of the BAILs on the cellulose hydrolysis to yield glucose.

## 2. Experimental

### 2.1 Materials

Microcrystalline cellulose (Avicel® PH-101, ∼50 μm particle size) was purchased from Sigma-Aldrich, Co. LLC. and dried under vacuum until constant weight before use. 1-Methylimidazole, 1-butylimidazole, 1,3-propanesultone, and 1-iodooctane were purchased from Wako Pure Chemical Industries, Ltd. and purified through distillation before use. Imidazole (>98.0%), chlorosulfonic acid (>97.0%), trifluoromethanesulfonic acid (TFS, >98.0%), methanesulfonic acid (MeS, >98.0%), benzenesulfonic acid (BzS, >98.0%), glacial acetic acid (AcOH, ≥99%), and 4-nitroaniline (>99.0%) were also purchased from Wako Pure Chemical Industries, Ltd. Potassium hydroxide (KOH, >86.0%), trifluoroacetic acid (TFAc, >99.0%), trichloroacetic acid (TClAc, 99%), sulfuric acid (H_2_SO_4_, >96.0%), and hydrochloric acid (HCl, 35.0–37.0%) were purchased from Kanto Chemicals Co., Inc. Bis(trifluoromethanesulfonyl)imide (HTFSI) was purchased from Morita Chemical Industries, Co., Ltd.; and phosphoric acid was purchased (>99.9%) from Sigma-Aldrich, Co. LLC. Other chemicals were also commercially available and used as received unless otherwise stated.

### 2.2 Apparatus

The ^1^H NMR spectra were recorded in DMSO-*d*_6_ on a ECX-300 spectrometer (JEOL, 7.05 T) operating at 300 MHz, and the chemical shifts are given in *δ* (ppm) downfield from TMS (*δ* = 0.00).

Cellulose hydrolysis assisted by microwave irradiation was carried out in a 2 mL borosilicate glass vial with a poly(ether ether ketone) (PEEK) cap and a Teflon-coated silicone septum (Anton Paar Japan, Co. Ltd.). Each reaction solution was heated in microwave irradiation equipment (MONOWAVE 300; Anton Paar Japan, Co. Ltd.) under the following conditions: frequency of 2.45 GHz (single mode), maximal setting output of 100 W, preheating time of 1 min, inner thermometer, ruby sensor (optic fibre), outer thermometer, and IR sensor.

Both the glucose assay and determination of *H*_0_ were conducted using a UV-vis spectrometer (UV-PC3100; Shimadzu Co.) and 10 mm micro quartz cells with a polytetrafluoroethylene (PTFE) stopper.

The FT-IR spectra of the samples, which were previously dried *in vacuo* for more than 24 h, were recorded on a Nicolet 6700 system with a MCT-A detector (Thermo Fisher Scientific, Inc., Tokyo, Japan) equipped with an ATR unit. The FT-IR measurements were conducted under a spectral resolution of 4 cm^−1^.

### 2.3 Synthesis of ILs

SO_3_H-functionalised BAILs were synthesised according to the literature,^25^ and as briefly explained below.

#### 2.3.1 Synthesis of Imds/HCl

Imidazole (6.81 g, 0.100 mol) was dissolved in 1,2-dichloroethane, and chlorosulfonic acid (12 mL, 0.200 mol) was added dropwise to the solution at ice bath temperature. After stirring for 12 h, the mixture was stood for 5 min to obtain a bi-layer solution. The resultant solution was washed with 1,2-dichloroethane repeatedly to obtain a white solid, namely 1,3-disulfonic acid imidazolium chloride (Imds/HCl). The crude product was washed with diethyl ether repeatedly, and the white solid was collected by vacuum filtration. The purified product was dried under vacuum for 24 h. Yield 59%, ^1^H NMR: (300 MHz; DMSO-*d*_6_, *δ*/ppm relative to TMS) *δ*: 10.70 (1H, br s), 9.10 (1H, d, *J* = 4.12 Hz), 7.70 (2H, d).

#### 2.3.2 Synthesis of Mims/HCl

1-Methylimidazole (8.20 g, 0.100 mol) was dissolved in 1,2-dichloroethane, and chlorosulfonic acid (7.2 mL, 0.120 mol) was added dropwise to the solution at ice bath temperature. After stirring for 20 min, the mixture was stood for 5 min to obtain a bi-layer solution. The resultant solution was washed with 1,2-dichloroethane repeatedly to obtain a viscous colourless liquid, namely 1-methyl-3-sulfonic acid imidazolium chloride (Mims/HCl). The crude product was purified through precipitation into diethyl ether, and the white solid was collected by vacuum filtration. The purified product was dried under vacuum for 24 h. Yield 81%, ^1^H NMR: (300 MHz; DMSO-*d*_6_, *δ*/ppm relative to TMS) *δ*: 9.06 (1H, s), 7.71 (1H, t), 7.66 (1H, t), 3.88 (3H, dt).

#### 2.3.3 Synthesis of Mimps, Bimps, and Oimps/HX

BAILs derived from three kinds of zwitterions were prepared according to the literature.^11,26^ An equivalent molar amount of acid (HX) was slowly added to the corresponding zwitterion (synthesised according to the literature^27,28^). The mixture was stirred at 80 °C for 3 days to obtain a viscous liquid.

For 1-(1-methyl-3-imidazolio)propane-3-sulfonate (Mimps), 1-methylimidazole (3.20 g, 39.0 mmol) was dissolved in aceto-nitrile, and 1,3-propanesultone (4.76 g, 39.0 mmol) was added dropwise to the solution. The mixture was stirred at r.t. for 3 days to obtain a white solid. Then, the crude product was washed with diethyl ether repeatedly, and further purified through recrystallisation with methanol twice. The purified product was dried under vacuum for 24 h. Yield 58%, ^1^H NMR: (300 MHz; DMSO-*d*_6_, *δ*/ppm relative to TMS) *δ*: 9.12 (1H, s), 7.78 (1H, t), 7.71 (1H, t), 4.30 (2H, t), 3.86 (3H, s), 2.41 (2H, t), 2.09 (2H, dt). Elemental analysis calcd (%) for C_7_H_12_N_2_O_3_S_1_: C, 41.2; H, 6.0; N, 13.7; S, 15.7, found: C, 41.1; H, 5.7; N, 13.7; S, 15.5.

For 1-(1-butyl-3-imidazolio)propane-3-sulfonate (Bimps), 1-butylimidazole (4.85 g, 39.1 mmol) was dissolved in acetonitrile, and 1,3-propanesultone (4.77 g, 39.1 mmol) was added dropwise to the solution. The mixture was stirred at r.t. for 3 days to obtain a white solid. Then, the crude product was washed with diethyl ether repeatedly, and further purified through recrystallisation with ethanol twice. The purified product was dried under vacuum for 24 h. Yield 82%, ^1^H NMR: (300 MHz; DMSO-*d*_6_, *δ*/ppm relative to TMS) *δ*: 9.21 (1H, s), 7.82 (2H, tt), 4.30 (2H, t), 4.18 (2H, t), 2.42 (2H, t), 2.11 (2H, dt), 1.79 (2H, dt), 1.27 (2H, dq), 0.91 (3H, t). Elemental analysis calcd for C_10_H_18_N_2_O_3_S_1_: C, 48.8; H, 7.4; N, 11.4; S, 13.0, found: C, 48.9; H, 7.3; N, 11.4; S, 13.2.

For 1-(1-octyl-3-imidazolio)propane-3-sulfonate (Oimps), imidazole (20.0 g, 0.294 mol) and KOH (25.2 g, 0.451 mol) were dissolved in acetonitrile and stirred at r.t. for 24 h. 1-Iodo-octane (35.2 g, 0.146 mol) was added dropwise to the solution. The mixture was refluxed at 80 °C for 12 h. After the removal of excess solvent using a rotary evaporator, the concentrated solution was washed with dichloromethane repeatedly to obtain a pale yellow liquid, namely 1-octylimidazole. The crude product was purified through distillation to obtain a colourless clear liquid. The purified 1-octylimidazole (9.01 g, 50.0 mmol) was dissolved in acetonitrile, and 1,3-propanesultone (6.11 g, 50.1 mmol) was added dropwise to the solution. The mixture was stirred at r.t. for 3 days to obtain a white solid. This crude product was washed with diethyl ether repeatedly. The purified product was dried under vacuum for 24 h. Yield 70%, ^1^H NMR: (300 MHz; DMSO-*d*_6_, *δ*/ppm relative to TMS) *δ*: 9.19 (1H, s), 7.80 (2H, tt, *J* = 8.93 Hz), 4.31 (2H, t), 4.16 (2H, t), 2.40 (2H, t), 2.09 (2H, dt), 1.78 (2H, m), 1.25 (10H, m), 0.86 (3H, dt). Elemental analysis calcd for C_14_H_26_N_2_O_3_S_1_: C, 55.6; H, 8.7; N, 9.3; S, 10.6, found: C, 55.6; H, 8.7; N, 9.2; S, 10.5.

#### 2.3.4 Synthesis of [Hmim]Cl

1-Methylimidazole (2.36 g, 14.4 mmol) and HCl (2.4 mL, 14.4 mmol) were mixed and stirred at 60 °C for 5 h to synthesise 1-methylimidazolium chloride ([Hmim]Cl). The crude product was purified by stirring with activated carbon in methanol at r.t. for 1 h. The resultant solution was filtrated to remove the activated carbon, and methanol was evaporated from the filtrate. The purified [Hmim]Cl was dried *in vacuo* for 24 h to obtain a white solid. Yield 68%, ^1^H NMR: (300 MHz; DMSO-*d*_6_, *δ*/ppm relative to TMS) *δ*: 3.85 (3H, dt), 7.78 (1H, t), 7.67 (1H, t), 9.14 (1H, s).

### 2.4 Determination of Hammett acidity function (*H*_0_)

The Brønsted acidity of the BAILs in water in terms of *H*_0_ was determined by UV-vis spectroscopy following the concept reported in the literature,^9,29^ using 4-nitroaniline as a basic indicator to receive the dissociative protons.

Upon increasing the acidity of the BAILs, the absorbance of the unprotonated form of the indicator (denoted as I) decreases, whereas its protonated form (IH^+^) could not be observed because of its low molar absorptivity. Therefore, the [I]/[IH^+^] ratio can be determined from the absorbance after the addition of BAILs. Then, *H*_0_ can be calculated using eqn (1), and it can be regarded as the relative proton donating ability of the BAILs in water.1
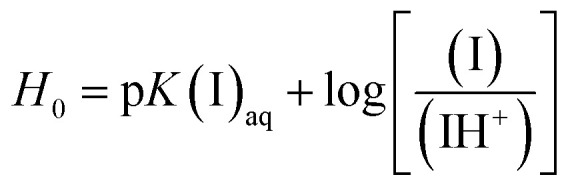


**Fig. 1:**
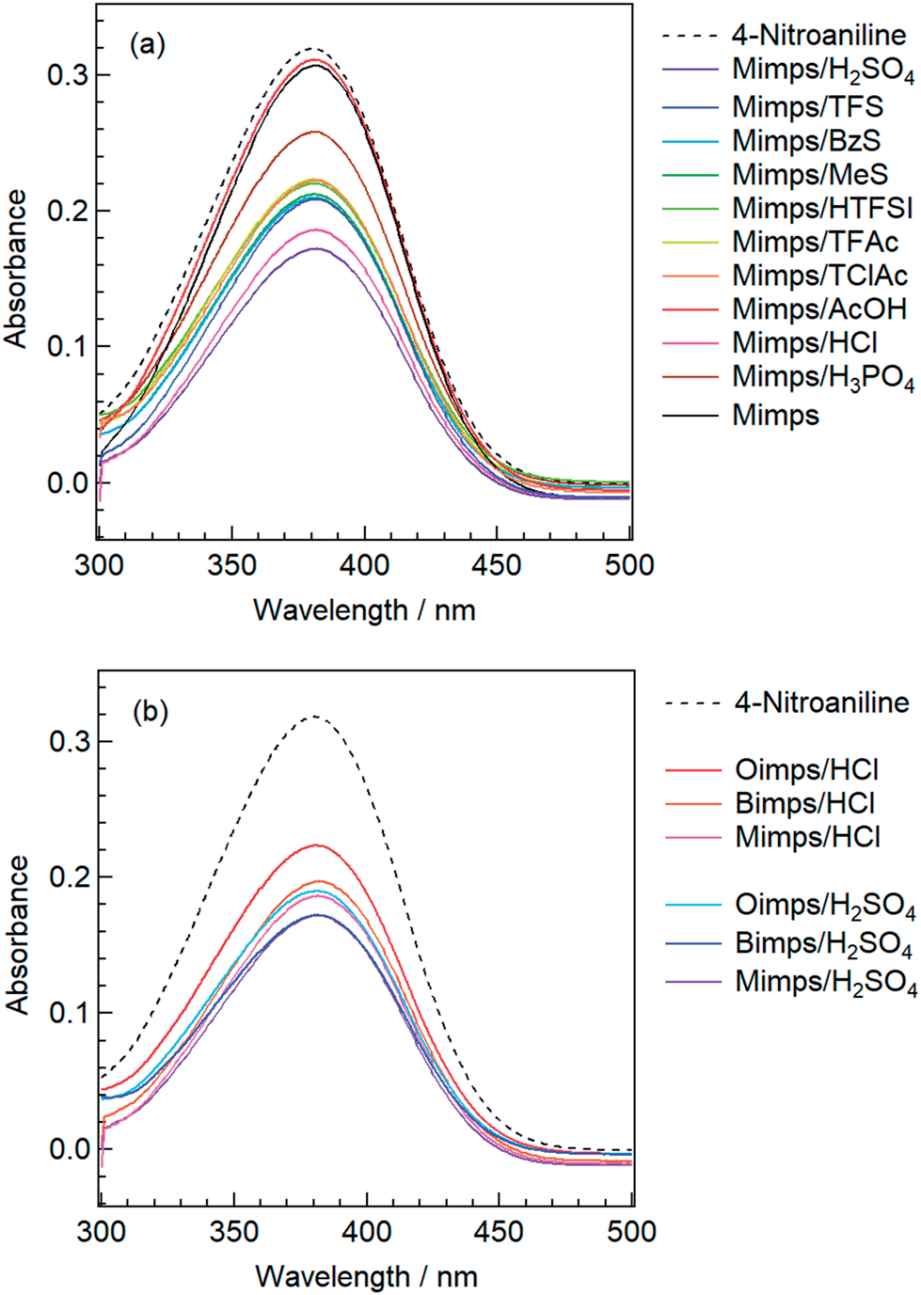
UV-vis spectra of 4-nitroaniline in water before and after the addition of BAILs with (a) various anions and (b) cations with different alkyl side chain lengths.

The *H*_0_ values of the BAILs were determined under the prescribed concentrations of 4-nitroaniline (3 mg L^−1^, p*K*(I)_aq_ = p*K*_a_ = 0.99) and BAIL (50 mmol L^−1^) in aqueous solution. The maximal absorbance of the I form of the indicator is observed at 380 nm in water. After the addition of BAILs into 4-nitroaniline aqueous solutions, the absorbance of the solutions decreased as shown in Fig. 1.

### 2.5 Molecular geometries

The minimum energy geometries of the BAILs were calculated by DFT optimisation at the B3LYP/6-311G++(d,p) level using the Gaussian 09 program series. The exact minimum was verified through the vibrational analysis of each optimised structure to ensure the absence of negative frequencies.^16,23,24^

### 2.6 Microwave-assisted hydrolysis of cellulose

An aqueous solution of BAIL (1 mol L^−1^) was prepared by adding the appropriate amount of deionised water. The accuracy of the solution concentration was checked by titration with a standardised 0.5 mol L^−1^ NaOH aqueous solution using phenolphthalein as an indicator.

Cellulose (10 mg) was added to a 1 M BAIL aqueous solution (1 mL) in a 4 mL borosilicate glass vial. The vial was firmly closed and heated for 15 min at 160 °C by microwave irradiation. The resultant solution was immediately cooled to ice bath temperature to quench the reaction, and neutralised by dropwise addition of 0.5 mol L^−1^ NaOH aqueous solution. The turbid solution was filtrated to remove the brownish residues. Then, the filtrate was collected for glucose assay.

### 2.7 Glucose analysis using a mutarotase/GOD method assay

The glucose yield after cellulose hydrolysis was determined through the mutarotase/glucose oxidase (GOD) method.^30^ The clear hydrolysate solution (20 μL) was transferred into a sample tube. At time 0, the enzymatic reaction was started by adding 4 mL of mutarotase/GOD assay reagent (Glucose CII test Wako kit; Wako Pure Chemical Industries, Ltd.) to the sample tube. The reaction solution was incubated in a water bath at 37 °C for 4 min to obtain a pink solution. Then, the absorbance of this solution at 505 nm was immediately measured by a UV-vis spectrometer. A reagent blank was prepared by the same treatment, using 20 μL of deionised water with 4 mL of assay reagent. The glucose concentration was calculated by employing a calibration curve obtained using the standard glucose solutions. The glucose yield was calculated according to eqn (2):2
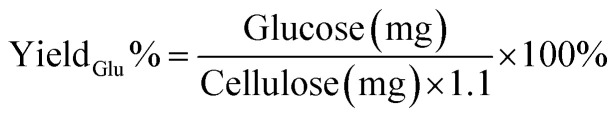
where the value of 1.1 was the molecular weight ratio between glucose (C_6_H_12_O_6_, 180 g mol^−1^) and one anhydrous glucose unit in cellulose (C_6_H_10_O_5_, 162 g mol^−1^).

## 3. Results and discussion

### 3.1 Acidic catalytic activity of SO_3_H-functionalised BAILs for cellulose hydrolysis

Firstly, the acidic catalytic activity of the SO_3_H groups in the BAILs and the effect of the spacer length between the SO_3_H group and imidazolium cation were investigated. Table 1 summarises the chemical structures of the BAILs (Imds/HCl, Mims/HCl, and Mimps/HCl), a zwitterion (Mimps), and a protic IL([Hmim]Cl), and the corresponding glucose yields. Both Mimps, which has a sulfonic acid group without an acidic proton, and [Hmim]Cl showed very low glucose yields (below 4%), suggesting poor acidic catalytic ability for cellulose hydrolysis, even though [Hmim]Cl has a proton in the imidazolium cation. The difference between the p*K*_a_ values (*i.e.*, Δp*K*_a_) of HCl (p*K*_a_ = −7.0) and 1-methylimidazole (p*K*_a_ = 7.06) is above 10, which may cause the originally active proton to stick on the imidazolium cation.^31^ On the other hand, all of the SO_3_H-functionalised BAILs showed higher glucose yields (over 20%), suggesting that the existence of the active proton in the SO_3_H group was key for the acidic catalytic activity of the BAILs.

**Table 1:** Acid catalytic abilities for cellulose hydrolysis and chemical structures of SO_3_H-functionalised BAILs with different spacer lengths between the SO_3_H groups and imidazolium cations, and a zwitterion and a protic IL as references

Entry	Chemical structure	Glucose yield (%)
Imds/HCl	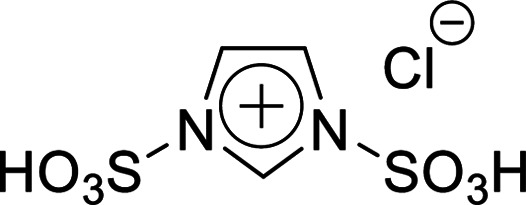	35 ± 0.3
Mims/HCl	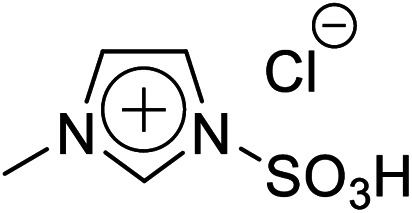	30 ± 2.3
Mimps/HCl	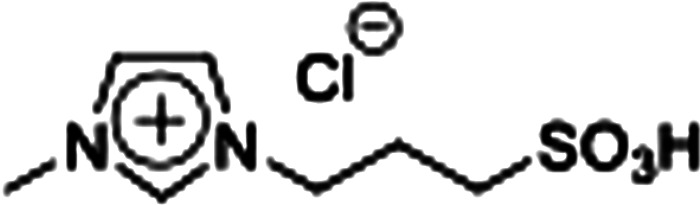	24 ± 0.3
Mimps	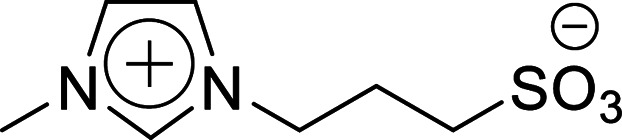	3.6 ± 0.3
[Hmim]Cl	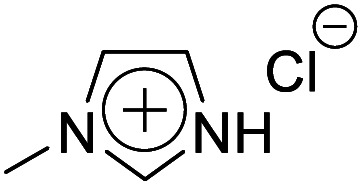	1.5 ± 1.0

BAILs with shorter spacer lengths and two SO_3_H groups showed higher glucose yields. The activity order of the BAILs for cellulose hydrolysis (Imds/HCl > Mims/HCl > Mimps/HCl) agrees with that for the hydration reaction of phenyl acetylene.^24^ Both Imds/HCl and Mims/HCl showed excellent acidic catalytic ability. However, the relatively low thermal and chemical stability of the corresponding zwitterions may be a concern (Fig. S1 and S2†). The lack of alkyl spacer may provide the ready desorption of SO_3_H groups with the release of chlorosulfonic acid, especially under basic conditions.^25^ Flexible alkyl spacers improve the stability of the zwitterions themselves, and also assist the approach of the SO_3_H group to the glycoside bond of cellulose. Moreover, it has been reported that BAILs with a propyl spacer (C3) exhibit higher TRS yields in cellulose hydrolysis than those with a butyl spacer (C4).^14,15,21^ According to these results, it was concluded that the SO_3_H-functionalised BAILs with a C3 spacer are the most suitable for investigating the detailed structural effects on the acidic catalytic activity of BAILs in cellulose hydrolysis.

### 3.2 Structural effects of BAILs on acidity and glucose yield

#### 3.2.1 Anion species

The *H*_0_ value in water was investigated for different BAILs with a Mimps cation and various anions (X^−^), abbreviated as Mimps/HX (Table 2). The HSO_4_ anion with an active proton in itself showed the lowest *H*_0_ value, and the Cl anion was the second lowest one. Neither Mimps/AcOH nor Mimp showed proton donation to the basic indicator as shown in Fig. 1(a), indicating that they hardly work as acidic catalysts.

**Table 2:** Calculation and comparison of the *H*_0_ values of BAILs with various anions and cations with different alkyl chain lengths in water at room temperature

Zw^a^	HX^b^	*A*_max_	I (%)	IH^+^ (%)	*H*_0_
None^c^	—	0.314	100	0	—
Mimps	H_2_SO_4_	0.172	55	45	1.07
	HCl	0.186	59	41	1.15
	TFS	0.208	66	34	1.28
	BzS	0.210	67	33	1.30
	MeS	0.212	68	32	1.31
	HTFSI	0.220	70	30	1.36
	TFAc	0.222	71	29	1.37
	TClAc	0.223	71	29	1.38
	H_3_PO_4_	0.258	82	18	1.65
	AcOH	0.311	99	1	2.96
	—	0.307	98	2	2.63
Bimps	H_2_SO_4_	0.186	59	41	1.14
	HCl	0.197	63	37	1.22
Oimps	H_2_SO_4_	0.197	62	38	1.21
	HCl	0.223	71	29	1.38

a Zw: zwitterion.

b HX: acid used to prepare the BAILs labelled as Zw/HX, where X corresponds to the anion species.

c Indicator: 4-nitroaniline.

**Fig. 2:**
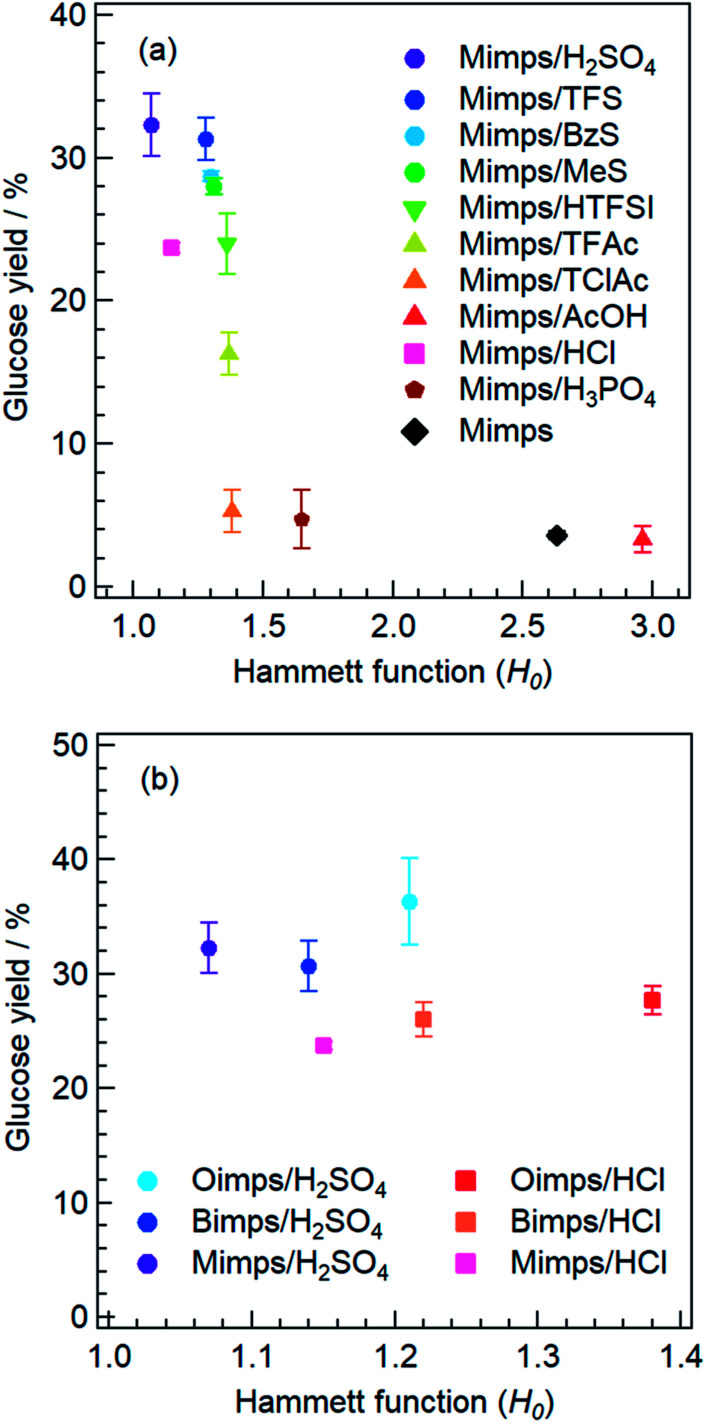
Correlation between the glucose yields and *H*_0_ values of BAILs with various anions (left) and different alkyl chain lengths of the cation (right).

Next, to determine the relationship between the *H*_0_ of the BAIL aqueous solutions and the acidic catalytic activity, these BAILs were used for cellulose hydrolysis, and the glucose yields were compared. Fig. 2(a) shows the relationship between the *H*_0_ of Mimps/HX and the glucose yield. Mimps/H_2_SO_4_ with the lowest *H*_0_ showed the highest glucose yield (32 ± 2.2%) among the BAILs used in this study. With increasing *H*_0_, the glucose yield tends to become lower. This result implies that the anion species of BAILs certainly affects their proton donor activity, which is related to the acidic catalytic activity in terms of glucose yield.

Consistent with their high *H*_0_ values, Mimps/AcOH and Mimps showed little acidic catalytic ability, and the glucose yields were 3.3 ± 0.9% and 3.6 ± 0.3%, respectively. Both Mimps/H_3_PO_4_ (4.7 ± 2.0%) and Mimps/TClC (5.3 ± 1.5%) also worked slightly as acidic catalysts for cellulose hydrolysis, although they had medium acidity. BAILs with the H_2_PO_4_ anion have also been reported to show poor acidic catalytic ability in cellulose hydrolysis with conventional heating, due to their low acidity.^21^ These results imply that, to function as an acid catalyst for cellulose hydrolysis, a BAIL needs to have a *H*_0_ lower than 1.5 at the least. Detailed discussion will be given below in Section 3.4 on estimating the acidic catalytic activity of BAILs using theoretical studies.

Notably, the glucose yield of Mimps/HCl (23.7 ± 0.3%) was lower (despite its second lowest *H*_0_) than that of BAILs with sulfonic acid anions, including BzS (28.7 ± 0.3%) and MeS (28.0 ± 0.6%). It was reported that BAILs with Cl anions are superior acidic catalysts compared to those with HSO_4_ in cellulose hydrolysis.^10,11,24^ However, the Cl anion-based BAILs significantly have been shown to accelerate not only cellulose hydrolysis, but also the subsequent dehydration reaction of the generated glucose to produce HMF.^10,21^ These serial reactions might cause a relatively low glucose yield. Under hydrothermal conditions at 120–160 °C, glucose is reversibly transformed first into fructose, and subsequently into HMF through dehydration reactions.^32^ Generally, the isomerisation of glucose to fructose occurs under a Lewis acid catalyst, but in the presence of Cl anion-based BAILs, the reactions go through the complexation of the BAILs with the open-chain sugars.^33^ While it is unclear whether similar dehydration reactions occur under other BAILs with different anions, a high Brønsted acidity is expected to lower the glucose yields through further conversion to HMF or other derivatives. The amount of residue after cellulose hydrolysis using Mimps/HCl was only one-fourth as compared with that of Mimps/H_2_SO_4_ as shown in Table S2,† suggesting the superior acidic catalytic ability of Mimps/HCl for this series of reactions.

#### 3.2.2 Alkyl side chain length of the cations

The effect of alkyl side chain length of the imidazolium cations on the *H*_0_ value in water was subsequently examined by UV-vis measurements. The changing absorbance of the basic indicator in water is shown in Fig. 1(b). Table 2 also summarises the *H*_0_ values of BAILs with methyl, butyl, and octyl groups on the cations in combination with Cl or HSO_4_ anions. Clearly, BAILs with an octyl group in the cation have higher *H*_0_ values regardless of the anion species.

The relationship between the *H*_0_ value and glucose yield for BAILs with different alkyl side chain lengths is depicted in Fig. 2(b). The glucose yields gradually improved with increasing alkyl side chain length, despite the concomitant increase in *H*_0_ value, which decreases the glucose yield when changing the anion species, as shown in Fig. 2(a). In a previous study of a similar cellulose conversion process, it was demonstrated that in dichloroethane, BAILs with longer alkyl side chains have higher *H*_0_ and lower HMF yields.^34^ This might be derived from the suppressed dehydration reaction of glucose by decreasing the proton donor activity of the BAILs. Thus, cellulose hydrolysis was semi-selectively promoted in BAILs with long alkyl side chains, increasing the glucose yield. The increased weight of the residual after cellulose hydrolysis using BAILs based on Bimps and Oimps cations also supports this conclusion (see Table S2†).

### 3.3 Estimation of acidic catalytic activity by considering the hydrogen bond network between BAILs

#### 3.3.1 Confirmation of cation–anion interaction using FT-IR

The above results show that the *H*_0_ value is an important index for evaluating the acidic catalytic activity of BAILs in water, and this value depends on both the anion species and alkyl side chain length of the cations. While there seems to be a certain relationship between *H*_0_ determined by the structures of the BAILs and glucose yields, a few exceptions were observed. For example, the glucose yield was relatively low for BAILs with the Cl anion despite their low *H*_0_ values, as shown in Fig. 2. Moreover, the relationship between glucose yield and *H*_0_ value for BAILs with various anion species (Fig. 2(a)) was different when changing the alkyl side chain length of the cations (Fig. 2(b)). Based on these specific observations, it can be said that the glucose yield does not depend solely on the *H*_0_ value.

**Fig. 3:**
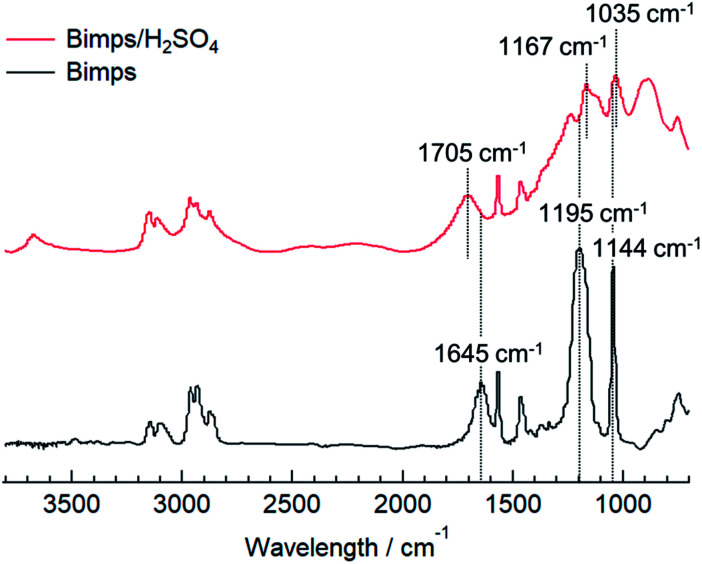
ATR mode FT-IR spectra of Bimps/H_2_SO_4_ (upper) and Bimps (lower) after drying *in vacuo* at 80 °C for 24 h.

From a different viewpoint, the hydrogen bond network between the cation and anion is also an important factor when evaluating the acidic catalytic activity of BAILs. To confirm the cation–anion interaction in the BAILs, the FT-IR spectrum of Bimps/H_2_SO_4_ as a representative BAIL was measured and compared to that of the pristine zwitterion (Bimps), as shown in Fig. 3. Considering the high hydrophilicity of the BAIL and zwitterion, both samples were carefully dried *in vacuo* just before the ATR mode FT-IR measurements. The stretching vibration bands of the SO_3_ group generally appear as two peaks at around 1200 and 1050 cm^−1^. The corresponding peaks appeared at 1195 and 1144 cm^−1^ in the spectrum of Bimps, and shifted to 1167 and 1035 cm^−1^ in that of Bimps/H_2_SO_4_, respectively. In addition, another peak derived from the stretching vibration of the N=C bond on the imidazolium ring was recorded at 1645 cm^−1^ for Bimps, and this peak shifted to 1705 cm^−1^ in the presence of H_2_SO_4_. These peak shifts strongly imply the existence of an interaction between the Bimps cation and HSO_4_ anion. In particular, the evident shifts of the peaks associated with the SO_3_ group clearly indicate that the proton derived from the used Brønsted acid was transferred onto the SO_3_ group in the zwitterion to afford the SO_3_H group in the BAILs. In addition, the shift of the N=C bond stretching peak strongly implies an environmental change around the imidazolium cation, due to some interactions with the counter anion, presumably *via* hydrogen bonding.

#### 3.3.2 Theoretical studies of the structural effect on the hydrogen bond network in the BAILs

**Fig. 4:**
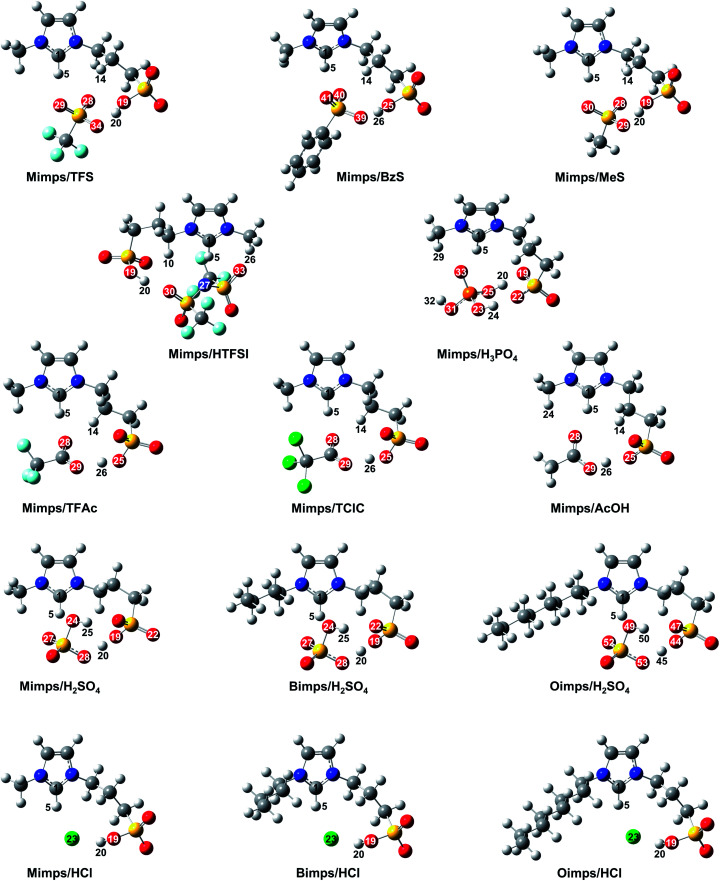
Optimised molecular structures of BAILs with various anions and different alkyl chain lengths in the cations calculated with B3LYP/6-311G++(d,p).

Despite the experimental confirmation of the cation–anion interaction in the BAILs, the FT-IR spectra only provide limited information about the detailed hydrogen bond network involved in the BAILs. Therefore, we further investigated the interaction between the ion pair in the BAILs through the minimum energy geometries, which were obtained through DFT geometry optimisations. The optimised structures of the BAILs are shown in Fig. 4 (see Fig. S3† for a more detailed version with labelling numbers on all atoms), and the geometry parameters are summarised in Table 3.

**Table 3:** Geometry parameters of the BAILs used in this study calculated with B3LYP/6-311G++(d,p)

Zw^a^	HX^b^	H–O bond of –SO_3_H (Å)	–SO_3_H⋯X (Å)	(N)_2_C–H bond (Å)	(N)_2_C–H⋯X (Å)	Other H.B. in BAILs (Å)
Mimps	H_2_SO_4_	H_20_–O_19_ = 1.073	H_20_–O_28_ = 1.392	C_1_–H_5_ = 1.091	H_5_–O_27_ = 1.853	H_25_–O_24_ = 0.980
					H_5_–O_24_ = 2.625	H_25_–O_22_ = 1.905
	HCl	H_20_–O_19_ = 1.008	H_20_–Cl_23_ = 2.051	C_1_–H_5_ = 1.103	H_5_–Cl_23_ = 2.167	—
	TFS	H_20_–O_19_ = 0.996	H_20_–O_34_ = 1.713	C_1_–H_5_ = 1.093	H_5_–O_29_ = 1.878	H_14_–O_28_ = 2.221
					H_5_–O_28_ = 2.850	
	BzS	H_26_–O_25_ = 1.004	H_26_–O_39_ = 1.632	C_1_–H_5_ = 1.086	H_5_–O_41_ = 1.984	H_14_–O_40_ = 2.483
					H_5_–O_40_ = 2.560	
	MeS	H_20_–O_19_ = 1.008	H_20_–O_29_ = 1.614	C_1_–H_5_ = 1.099	H_5_–O_30_ = 1.828	H_14_–O_28_ = 2.176
					H_5_–O_28_ = 2.823	
	HTFSI	H_20_–O_19_ = 0.993	H_20_–O_30_ = 1.701	C_1_–H_5_ = 1.092	H_5_–N_27_ = 1.978	H_26_–O_33_ = 2.148
						H_10_–O_30_ = 2.308
	TFAc	H_26_–O_25_ = 1.034	H_26_–O_29_ = 1.524	C_1_–H_5_ = 1.097	H_5_–O_28_ = 1.764	H_14_–O_28_ = 2.226
	TClAc	H_26_–O_25_ = 1.033	H_26_–O_29_ = 1.523	C_1_–H_5_ = 1.096	H_5_–O_28_ = 1.766	H_14_–O_28_ = 2.218
	H_3_PO_4_	H_24_–O_22_ = 1.640	H_24_–O_23_ = 1.002	C_1_–H_5_ = 1.095	H_5_–O_33_ = 1.814	H_32_–O_31_ = 0.963
		H_20_–O_19_ = 1.663	H_20_–O_25_ = 1.000			H_29_–O_33_ = 2.386
	AcOH	H_26_–O_25_ = 1.494	H_26_–O_29_ = 1.037	C_1_–H_5_ = 1.089	H_5_–O_28_ = 1.869	H_14_–O_28_ = 2.878
						H_24_–O_28_ = 2.627
Bimps	H_2_SO_4_	H_20_–O_19_ = 1.076	H_20_–O_28_ = 1.385	C_1_–H_5_ = 1.089	H_5_–O_27_ = 1.879	H_25_–O_24_ = 0.980
					H_5_–O_24_ = 2.635	H_25_–O_22_ = 1.913
	HCl	H_20_–O_19_ = 1.009	H_20_–Cl_23_ = 2.038	C_1_–H_5_ = 1.101	H_5_–Cl_23_ = 2.180	—
Oimps	H_2_SO_4_	H_45_–O_44_ = 1.073	H_45_–O_53_ = 1.392	C_1_–H_5_ = 1.089	H_5_–O_52_ = 1.885	H_50_–O_49_ = 0.979
					H_5_–O_49_ = 2.642	H_50_–O_47_ = 1.916
	HCl	H_20_–O_19_ = 1.009	H_20_–Cl_23_ = 2.042	C_1_–H_5_ = 1.101	H_5_–Cl_23_ = 2.186	—

a Zw: zwitterion.

b HX: acid used to prepare the BAILs labelled as Zw/HX, where X is the corresponding anion species.

The theoretical studies reveal the existence of hydrogen bond networks between the ion pair in the BAILs. Their acidic catalytic activity depends on the active proton in the SO_3_H group, a weak acidic proton at the C2 position of the imidazolium cation, and in the case of the HSO_4_ anion also the proton within the anion itself.^23^ However, the p*K*_a_ value of the acidic proton in the imidazolium ring is expected to be significantly higher than that of the SO_3_H group.^35^ Thus, the active proton in the SO_3_H group might be the main contributor to the acidic catalytic activity of the BAILs. The H–O bond distance in the SO_3_H group is shortened with weaker cation–anion interaction in the BAILs, which allows better accessibility to the substrate due to the higher mobility of the acidic proton in the SO_3_H group. Therefore, BAILs with a shorter H–O bond distance in the SO_3_H group are considered to have higher acidic catalytic activity. In the calculation results for BAILs with the Mimps cation, Mimps/HTFSI showed the shortest bond distance (H_20_–O_19_ = 0.993 Å), and the other BAILs with relatively short H–O distances can be ranked as Mimps/TFS (H_20_–O_19_ = 0.996 Å) < Mimps/BzS (H_26_–O_25_ = 1.004 Å) < Mimps/HCl (H_20_–O_19_ = 1.008 Å) ∼ Mimps/MeS (H_20_–O_19_ = 1.008 Å). Mimps/HTFSI was therefore expected to have the highest acidic catalytic activity according to the theoretical calculations. However, its actual glucose yield (24.0 ± 2.1%) was lower than that of BAILs with sulfonic acid anions (Fig. 2(a)). One explanation is that in this study, the cellulose hydrolysis experiments were conducted in an aqueous medium, and the DFT calculations failed to fully consider the effect of water molecules. Thus, Mimps/HTFSI may not fully realise its acidic catalytic ability in water because of the hydrophobicity of the TFSI anion,^36,37^ which may also be reflected in its relatively high *H*_0_ value (Table 2).

Despite it having the lowest *H*_0_, the H–O bond distance in the SO_3_H group of Mimps/H_2_SO_4_ (H_20_–O_19_ = 1.073 Å) was longer than that of Mimps/TFAc (H_26_–O_25_ = 1.034 Å) and Mimps/TClAc (H_26_–O_25_ = 1.033 Å), which showed much higher *H*_0_ values as described in Table 2. Mimps/H_2_SO_4_ has a significant hydrogen bond (H_25_–O_22_ = 1.905 Å) between the HSO_4_ anion and SO_3_H group (Fig. 4), and this strong hydrogen bond network would cause a longer H–O bond length in the SO_3_H group. However, Mimps/HSO_4_ has another acidic proton in the HSO_4_ anion. The oxygen of the HSO_4_ anion forms comparatively strong hydrogen bonding with the imidazolium ring hydrogen (H_5_–O_27_ = 1.853, H_5_–O_24_ = 2.625), which makes the proton in the HSO_4_ anion more labile and acidic.^23^ Therefore, in the particular case of BAILs with HSO_4_ anions, the active proton in the HSO_4_ anion might contribute to the acidic catalytic activity, in addition to the proton of the SO_3_H group that is the main source of acidic catalytic activity for other BAILs.

Hydrogen bond networks were also confirmed in BAILs with other sulfonic acid anions. The distance between the anion and SO_3_H group becomes shorter with increasing degree of hydrogen bonding. Such cation–anion interactions may inhibit the accessibility of the proton of the SO_3_H group to the glycoside bonds of cellulose for hydrolysis. Therefore, the actual acidic catalytic activities of Mimps/TFS and Mimps/BzS as shown in Fig. 2(a) were less than that of Mimps/H_2_SO_4_, as the latter also has the advantage of another active proton in the anion, regardless of the strength of the hydrogen bond networks.

Another hydrogen bond between the anion and the weak acidic proton at the C2 position of the imidazolium cation was observed. When the proton is closely covered by the anion, its accessibility is also reduced.^9^ Moreover, the shorter (N)_2_C–H distance due to the strong hydrogen bonding also makes the acidic proton less labile.^24^ However, these hydrogen bond networks are all absent in Mimps/HCl. The Cl anion is located in the middle between the SO_3_H proton (H_5_–Cl_23_ = 2.167 Å) and the one at the C2 position of the imidazolium cation (H_20_–Cl_23_ = 2.051 Å). Hence, in addition to the SO_3_H group being the main acid functional site, the weak acidic proton on the imidazolium ring also contributes to the acidic catalytic ability of Mimps/HCl. These structural advantages may provide superior acidic catalytic ability and accelerate both cellulose hydrolysis and glucose dehydration.

Despite the long alkyl chains in their cations, significant hydrogen bond networks were not observed in either Bimps/HCl or Oimps/HCl. On the other hand, Bimps/H_2_SO_4_ (H_25_–O_22_ = 1.913 Å) and Oimps/H_2_SO_4_ (H_50_–O_47_ = 1.916 Å) showed strong hydrogen bond networks, and the strength was slightly weaker for the longer alkyl side chain. It should be noted that the bond distances of both O–H in the SO_3_H groups (Zw/HCl: 1.008–1.009 Å, Zw/H_2_SO_4_: 1.073–1.076 Å) and C–H at the C2 position of the imidazolium cations (Zw/HCl: 1.101–1.103 Å, Zw/H_2_SO_4_: 1.089–1.091 Å) were quite constant. In general, *N*-methyl group substitution increases the electron density of the C2 carbon on the imidazolium ring, owing to the electron donating nature of the methyl group. However, the effect of the alkyl side chain length on the acidic catalytic ability of BAILs seemed to be slight, considering the small changes in the (N)_2_C–H bond distance in Table 3. This suggests that the alkyl side chain length of the imidazolium cations hardly affects the acidic catalytic activity of BAILs, if not considering the water effect.

However, higher *H*_0_ values in water were observed with longer alkyl side chains, as shown in Table 2. Such conflicting results imply that lengthening the alkyl side chains would increase the hydrophobicity of BAILs and thereby inhibit their acidic catalytic activity in cellulose hydrolysis in aqueous media. Therefore, it is expected that the dehydration of glucose was suppressed, which improves the glucose yield. In the cases of BAILs having both long alkyl chains (such as octyl (C8)) with high hydrophobicity and hydrophilic SO_3_H groups, their aggregate morphologies in water are expected to change.^38,39^ Such differences in the aggregation states of BAILs may also affect the cellulose hydrolysis in an aqueous medium, and detailed studies of this are in progress.

## 4. Conclusions

SO_3_H-functionalised BAILs with various structures were used as acid catalysts for cellulose hydrolysis assisted by microwave irradiation. The *H*_0_ value is a convenient method to evaluate the Brønsted acidity of BAILs in water. BAILs should have *H*_0_ < 1.5 to function as acidic catalysts, and there was a certain correlation between their *H*_0_ values in water and their acidic catalytic activity for cellulose hydrolysis. However, a long alkyl side chain in the cation, which increases hydrophobicity, increases the *H*_0_ value, but the glucose yield improved. Then, minimum energy geometries determined by *ab initio* calculations were used to help with the evaluation. The theoretical studies assessed the accessibility of multiple acidic protons in the BAILs, by considering anion–cation interactions that could hinder the mobility of acidic protons. Such interaction between the ion pair of the BAILs was also experimentally confirmed using IR spectroscopy. The strong hydrogen bond network in BAILs might inhibit the acidic catalytic activity, especially in the case of sulfonic acid anions, while the long alkyl chain had no effect. The moderate decrease of the Brønsted acidity of the BAILs in water is key for improving the glucose yield.

## Conflicts of interest

The authors declare no conflict of interest.

## Supplementary Material

RA-008-C8RA01950A-s001
