# Supplementary material for: Brønsted acidic ionic liquids for cellulose hydrolysis in an aqueous medium: structural effects on acidity and glucose yield
Source: RSC Adv. 2018 Apr 18;8(26):14623–32. doi: 10.1039/c8ra01950a (PMC9079954; doi:10.1039/c8ra01950a)
Supplement: RA-008-C8RA01950A-s001 [file RA-008-C8RA01950A-s001.pdf]

Electronic Supplementary Material (ESI) for RSC Advances.
This journal is © The Royal Society of Chemistry 2018

Electronic Supplementary Information (ESI)

Brønsted acidic ionic liquids for cellulose hydrolysis in aqueous medium: Structural effects on acidity and glucose yield

Shiori Suzuki,[#] Yuko Takeoka, Masahiro Rikukawa and Masahiro Yoshizawa–Fujita*

Department of Materials and Life Sciences, Sophia University, 7-1 Kioi–cho, Chiyoda–ku, Tokyo 102-8554, Japan. E-mail: masahi-f@sophia.ac.jp*

[#] Now at Faculty of Natural System, Institute of Science and Engineering, Kanazawa University, Kakuma-machi, Kanazawa 920-1192, Japan.

The file includes: Supporting Fig. S1–S3 and Table S1–S2

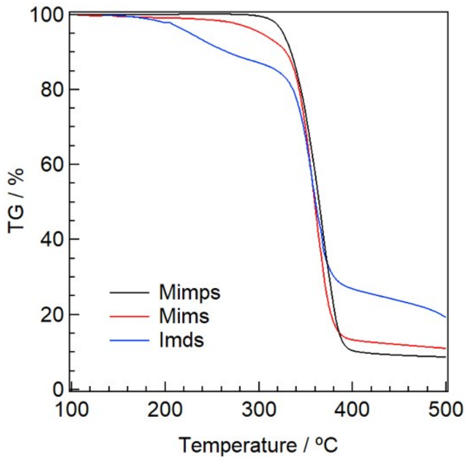

· Instrument: TG-DTA 7200 (Hitachi High-Technologies, Co. Ltd.)

· Apparatus: open-type aluminum pan

· Sample loading: 10 mg

· Heating rate: 10 ^{o}C min^{-1}

· N_2 flow: 200 mL min^{-1}

Fig. S1 TG curves of zwitterions.

Table S1 Thermal property of zwitterions

Zwitterion [a]	$T_{d\text{-}5\%}$ [b] / ^{o}C
Mimps	326
Mims	302
Imds	226

[a] Prior to TG measurement, each zwitterion was dried at 120°C for 1 h under N_2.
[b] $T_{d\text{-}5\%}$: 5% weight loss temperature.

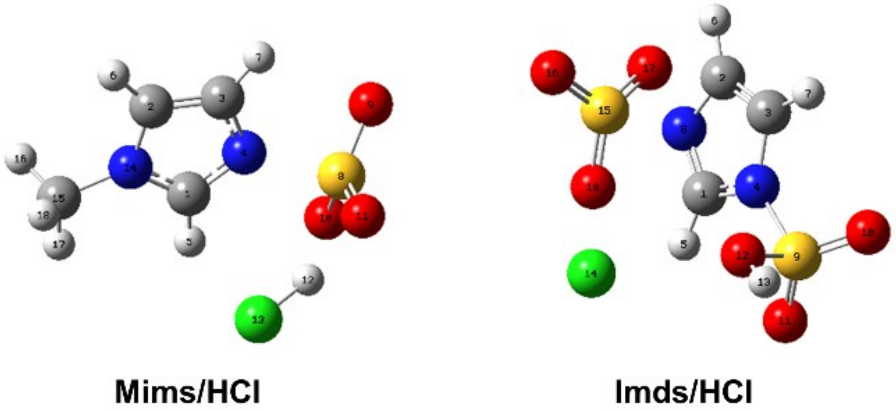

Fig. S2 Optimized molecular structures of BAILs with mono– or disulfuric acid groups using B3LYP/6-311G++(d, p).

Table S2 Residual amount of residue after cellulose hydrolysis (160 °C, 15 min) in 1 M BAILs aqueous solution

Zw [a]	HX [b]	Glucose yield / %	Residue [c] / *wt*.%
Mimps	H_2SO_4	32.3±2.2	5.6
Bimps		30.7±2.2	19
Oimps		36.3±3.8	21
Mimps	HCl	23.7±0.3	24
Bimps		26.0±1.5	30
Oimps		27.7±1.2	26

[a] Zw: zwitterion. [b] HX: kinds of acid used to prepare BAILs called as Zw/HX; X is corresponded to anion species. [c] Represented as *wt*.% to the starting weight of cellulose (10 mg).

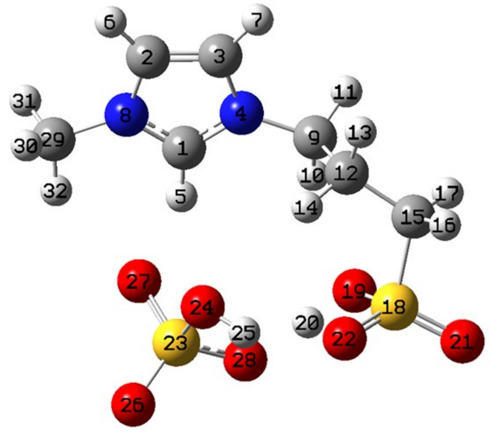

Mimps/H_2SO_4

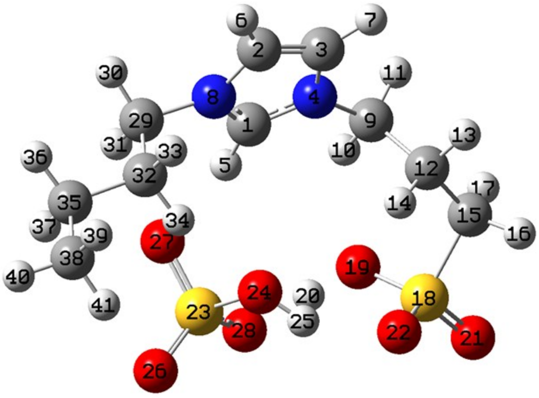

Bimps/H_2SO_4

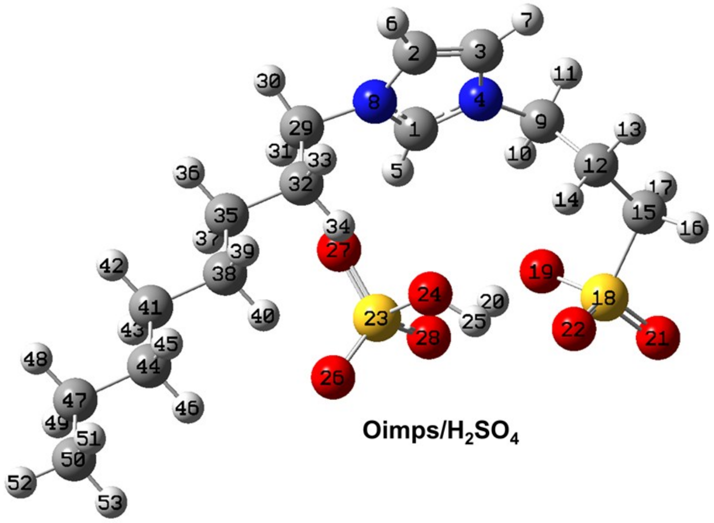

Oimps/H_2SO_4

Fig. S3 (1) Optimized molecular structures of BAILs with HSO_4 anion using B3LYP/6-311G++(d, p).

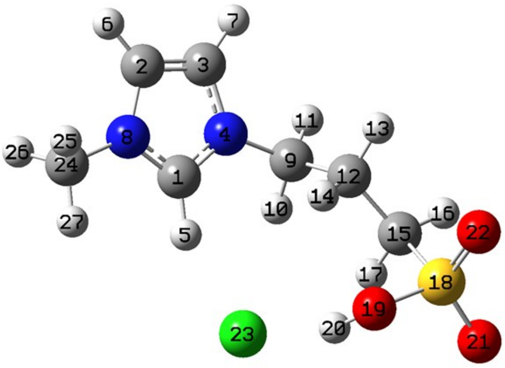

Mimps/HCl

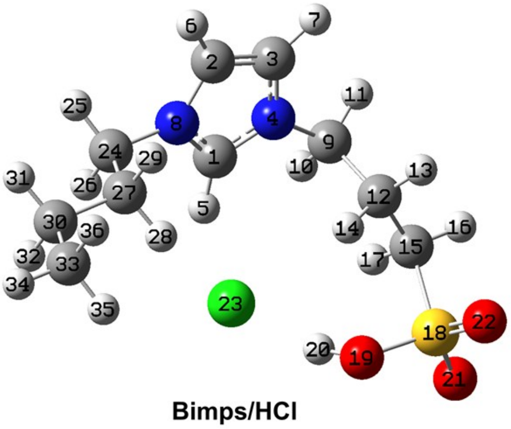

Bimps/HCl

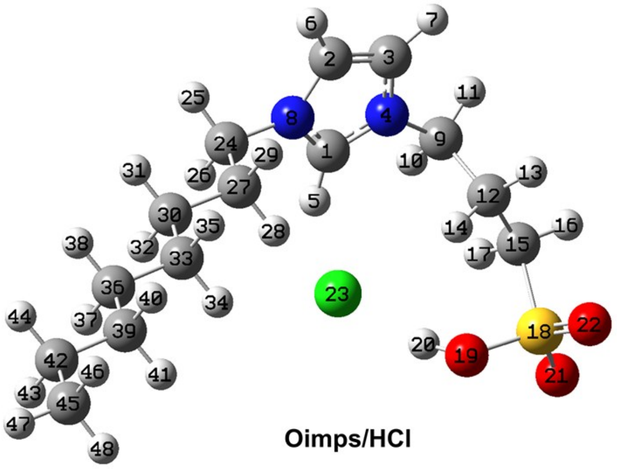

Oimps/HCl

Fig. S3 (2) Optimized molecular structures of BAILs with Cl anion using B3LYP/6-311G++(d, p).

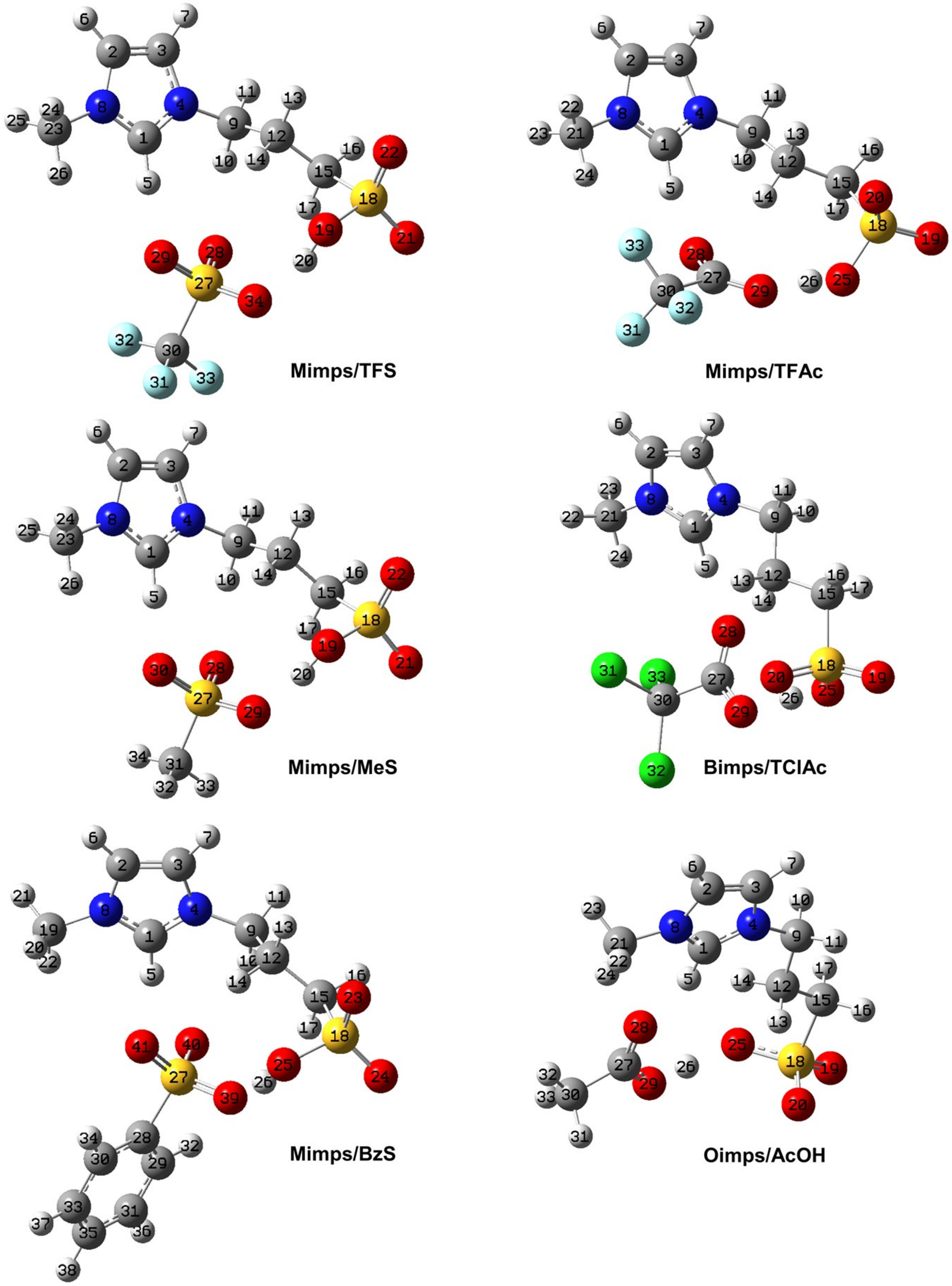

Fig. S3 (3) Optimized molecular structures of BAILs with sulfonic acid anon or carboxylic acid anion using B3LYP/6-311G++(d, p).

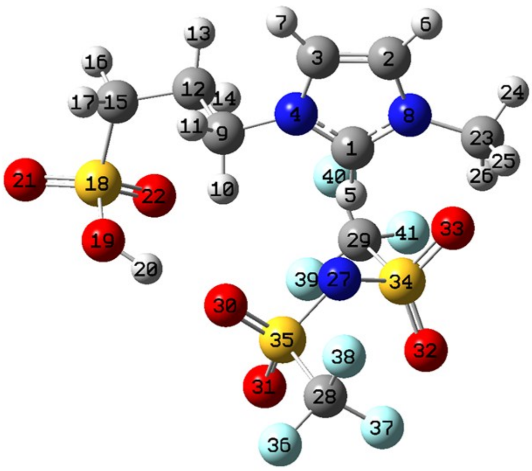

Mimps/HTFSI

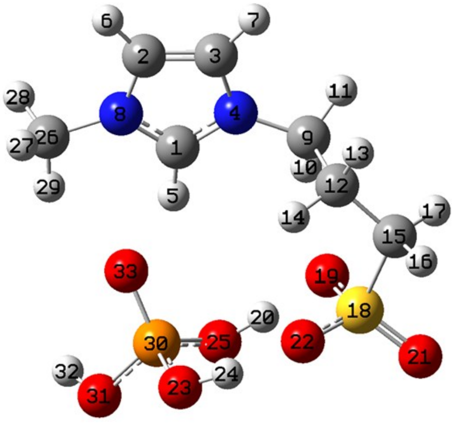

Mimps/H_3PO_4

Fig. S3 (4) Optimized molecular structures of BAILs with TFSI anon or phosphoric acid anion using B3LYP/6-311G++(d, p).
